# CERAMENT treatment of fracture defects (CERTiFy): protocol for a prospective, multicenter, randomized study investigating the use of CERAMENT™ BONE VOID FILLER in tibial plateau fractures

**DOI:** 10.1186/1745-6215-15-75

**Published:** 2014-03-08

**Authors:** Thomas Nusselt, Alexander Hofmann, Daniel Wachtlin, Stanislav Gorbulev, Pol Maria Rommens

**Affiliations:** 1 BiomaTiCS Group, Center for Orthopedics and Trauma Surgery, University Medical Center, Langenbeckstr. 1, Mainz D-55131, Germany; 2 Interdisziplinäres Zentrum Klinische Studien (IZKS), University Medical Center, Langenbeckstr. 1, Mainz D-55131, Germany

**Keywords:** bone graft, bone substitute, bone void, fracture, quality of life, pain

## Abstract

**Background:**

Bone graft substitutes are widely used for reconstruction of posttraumatic bone defects. However, their clinical significance in comparison to autologous bone grafting, the gold-standard in reconstruction of larger bone defects, still remains under debate. This prospective, randomized, controlled clinical study investigates the differences in pain, quality of life, and cost of care in the treatment of tibia plateau fractures-associated bone defects using either autologous bone grafting or bioresorbable hydroxyapatite/calcium sulphate cement (CERAMENT™|BONE VOID FILLER (CBVF)).

**Methods/Design:**

CERTiFy (CERament™ Treatment of Fracture defects) is a prospective, multicenter, controlled, randomized trial. We plan to enroll 136 patients with fresh traumatic depression fractures of the proximal tibia (types AO 41-B2 and AO 41-B3) in 13 participating centers in Germany. Patients will be randomized to receive either autologous iliac crest bone graft or CBVF after reduction and osteosynthesis of the fracture to reconstruct the subchondral bone defect and prevent the subsidence of the articular surface. The primary outcome is the SF-12 Physical Component Summary at week 26. The co-primary endpoint is the pain level 26 weeks after surgery measured by a visual analog scale. The SF-12 Mental Component Summary after 26 weeks and costs of care will serve as key secondary endpoints. The study is designed to show non-inferiority of the CBVF treatment to the autologous iliac crest bone graft with respect to the physical component of quality of life. The pain level at 26 weeks after surgery is expected to be lower in the CERAMENT bone void filler treatment group.

**Discussion:**

CERTiFy is the first randomized multicenter clinical trial designed to compare quality of life, pain, and cost of care in the use of the CBVF and the autologous iliac crest bone graft in the treatment of tibia plateau fractures. The results are expected to influence future treatment recommendations.

**Trial registration number:**

ClinicalTrials.gov: NCT01828905

## Background

### Background and rationale

Tibial plateau fractures (TPF) are caused by both low-energy or high-energy excessive varus or valgus forces combined with axial stress on the knee [1]. High-energy fractures are commonly the result of traffic accidents, falls, or sports-related injuries. Low-energy fractures are mainly seen in older individuals due to reduced bone mineral density and are typically associated with a depression of the articular surface. In all cases, depressed articular components require meticulous anatomical reduction and stable osteosynthesis to allow early rehabilitation. The depression of the articular surface may be associated with a metaphyseal defect, which often requires additional reconstruction procedures with autologous bone grafts or bone graft substitutes to improve healing and prevent subsidence of the articular surface [2]. Due to its favorable biological and biomechanical features [3-5], autologous iliac bone grafting (AIBG) has been considered a gold standard especially to reconstruct bone voids of large sizes and fracture non-unions even in metaphyseal and diaphyseal areas. However, some well-recognized complications associated with graft harvesting including pain at the donor site, nerve injury, hematoma, infection and pelvic fracture at the donor site [6-8] may significantly influence the quality of life (QoL) of those patients [9]. In addition, both prolonged operation time and hospital stay after bone graft harvesting may unnecessarily complicate the surgical treatment [9,10].

To address such shortcomings and to develop a non-invasive alternative to AIBG, the quality of synthetic bone graft substitutes has been increasingly improved in the last two decades [11]. In a non-controlled cohort study with a mean follow-up of 22.2 months bone union was achieved in all patients and remodeling of the bone substitute in 89% in depressed tibia plateau fractures [12]. In a prospective, randomized study on 119 patients with tibia plateau fractures treated with a bioresorbable calcium phosphate cement or autologous iliac bone graft the authors showed comparable union rates and times to union in the two groups. Interestingly, a significantly higher rate of articular subsidence was observed in the bone graft group [13].

Other studies on this topic focused on clinical and functional outcomes [14]. Bioactive glass has been compared to autologous bone in a randomized clinical study for the treatment of tibia plateau fractures. This study with 25 patients found the same clinical outcome in the two groups [15]. Clinical and functional outcome parameters have been investigated to compare autologous bone grafts and bone graft substitutes in several studies. However, the endpoints used in these studies were strongly related to the quality of the surgery rather than to the quality of the bone graft material itself. None of the published studies investigated parameters such as pain, quality of life or cost of care, which may really reflect the consequences of the bone harvesting. On the other hand these parameters are also suitable to assess the effects of the bone substitute material quality, which is critical for the defect healing and prevention of the secondary subsidence of the joint surface. Therefore, investigation of these outcome parameters in a sufficiently powered, prospective, randomized, controlled clinical trial is highly necessary.

**Figure 1:**
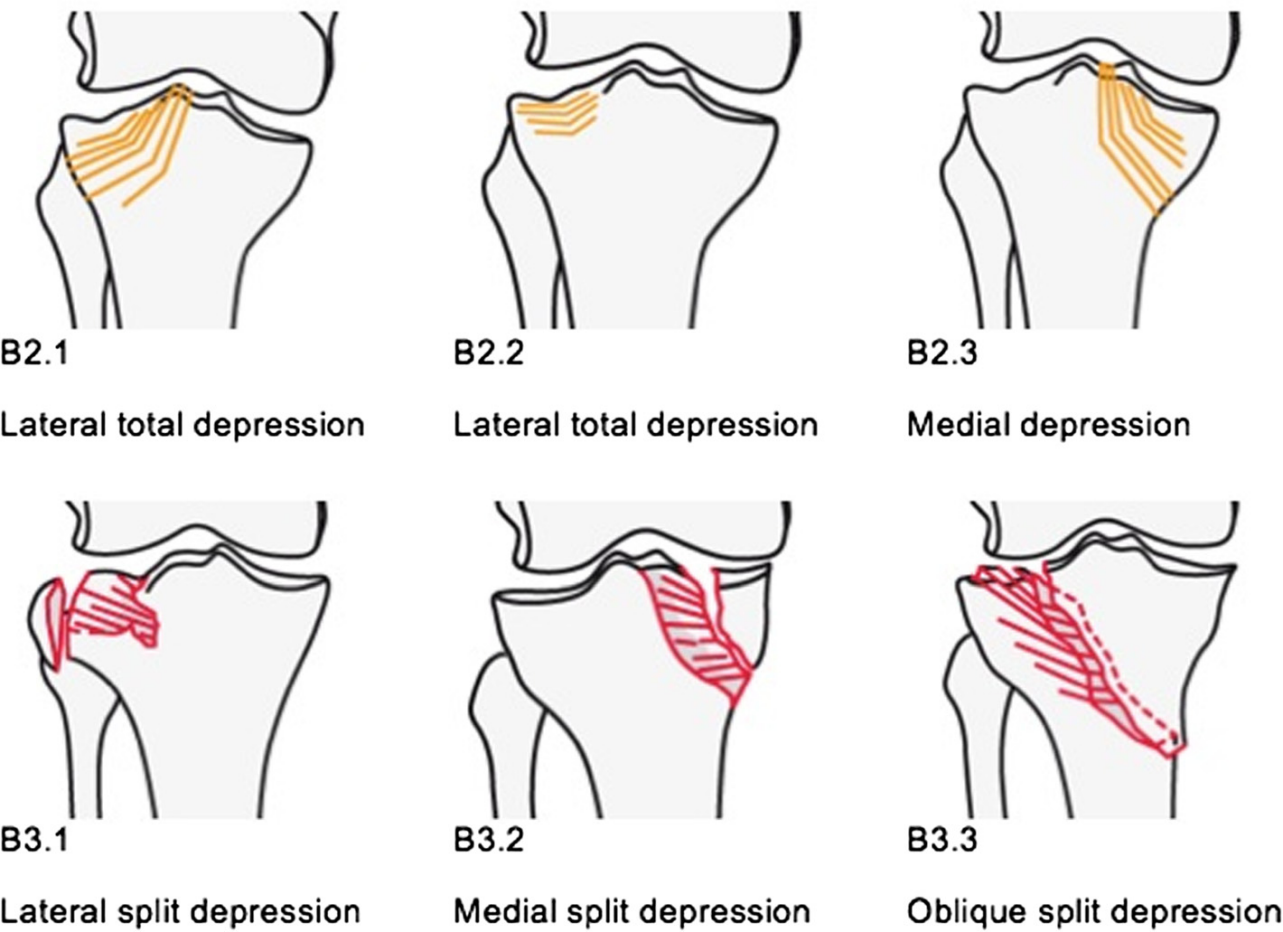
**AO classification of tibia plateau fractures (B2 and B3).** (‘Copyright by AO Foundation, Davos, Switzerland - Müller AO Classification of Fractures’).

CERAMENT™ BONE VOID FILLER (CBVF) is one of the widely used bone substitute materials, which was reported to promote cancellous bone healing and reproducible remodeling in bone defects [16]. It consists of 60% calcium sulfate (CS) and 40% hydroxyapatite (HA) and has been previously investigated in an open wedge radius osteotomy study showing convincing results. The aim of this study is to compare the impact of CBVF and autologous bone graft on QoL, pain, and costs of care in a standardized tibial plateau fracture (AO-Types 41-B2 and AO 41-B3 (Figure 1)) that are associated with a significant bone defect.

### Primary hypothesis

We assume that six months after surgical treatment no relevant mean differences will be present for the SF-12-PCS (Short Form 12 Physical Component Summary Score) and that pain level (determined by Visual Analog Score, VAS) will be lower in patients treated with CBVF compared to autologous cancellous bone grafts in tibia plateau fractures types AO 41-B2 and AO 41-B3.

## Methods/Design

The CERTiFy (CERament treatment of Tibial Fracture defects) trial is a prospective, open-label, multicenter, controlled, randomized clinical trial. The study will comprise 136 patients with acute traumatic depression fractures of the proximal tibia (limited to AO type 41-B2 & B3) from 13 different trauma centers in Germany. Patients are randomized to receive either an autologous cancellous bone graft taken from the iliac crest or CBVF.

### Eligibility criteria

**Figure 2:**
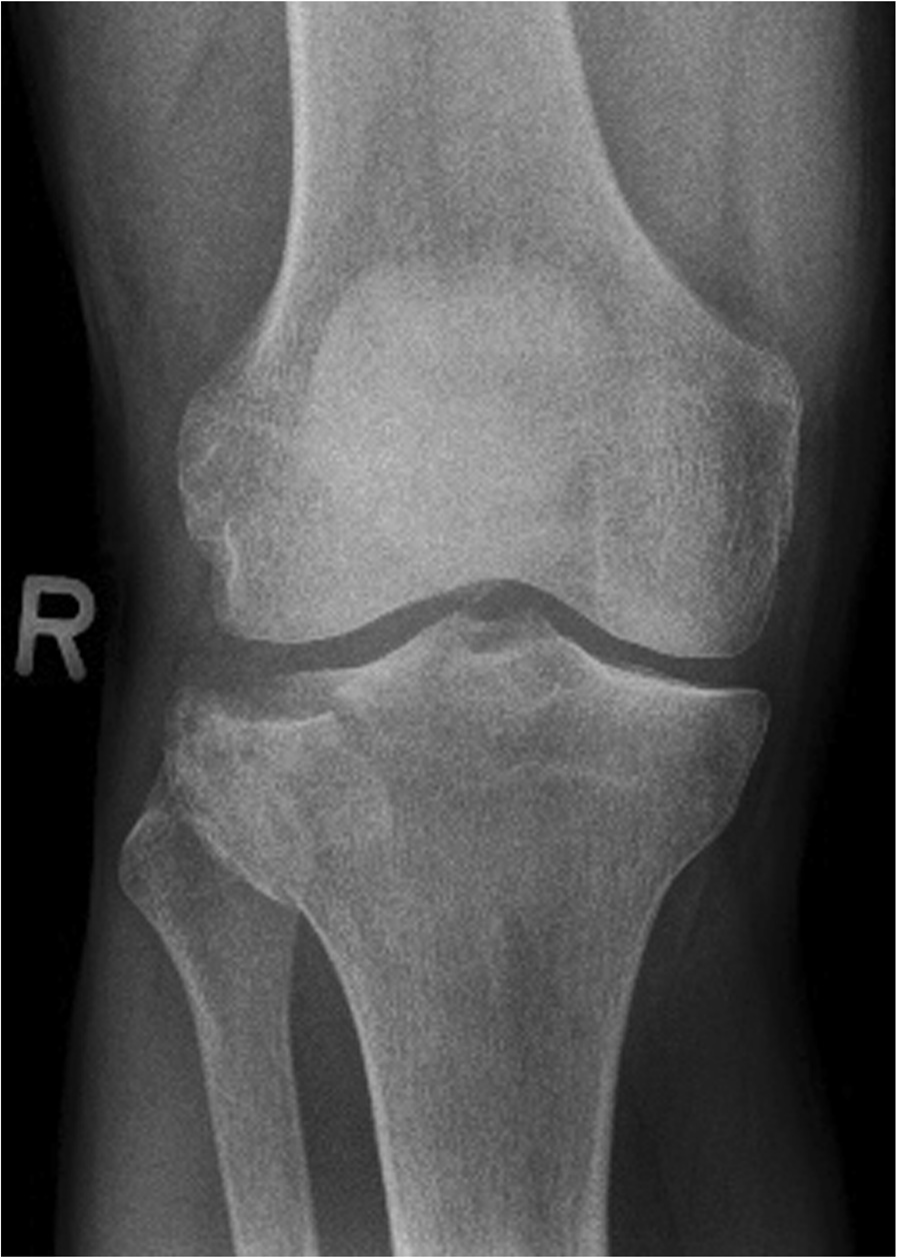
Anteroposterior (a-p) X-ray of the knee showing an AO 41 B3.1 fracture type.

**Figure 3:**
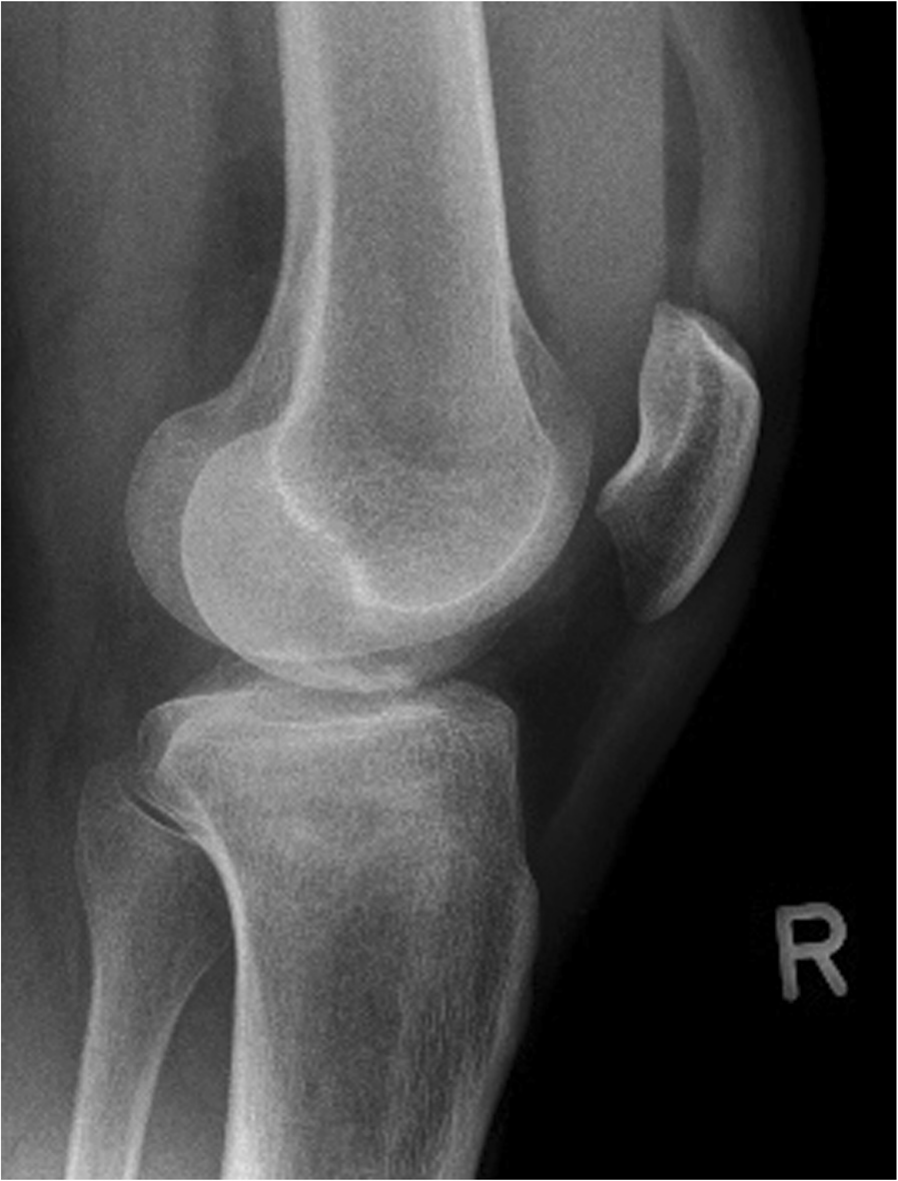
Lateral X-ray of the knee showing an AO 41 B3.1 fracture type.

**Figure 4:**
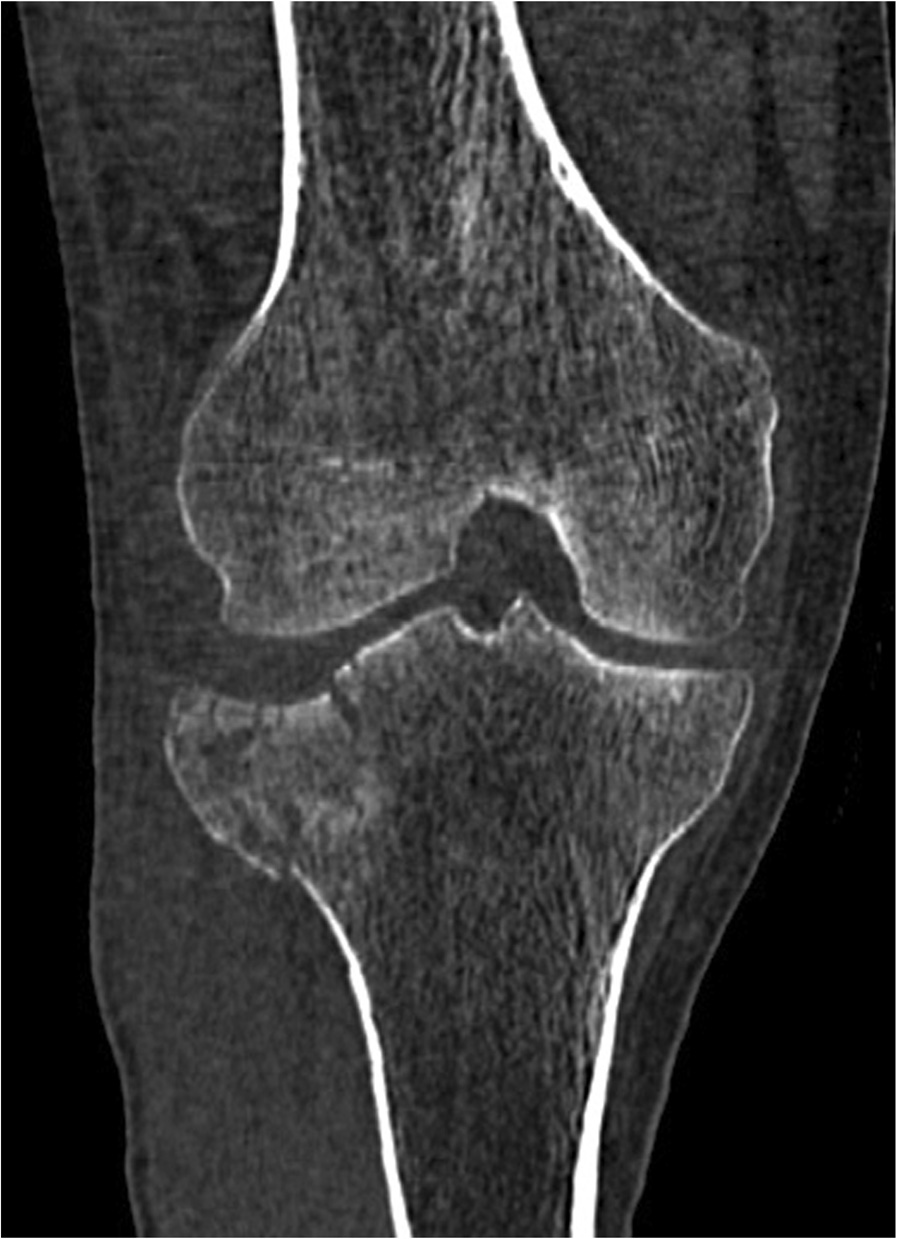
Coronal reconstructions of a computed tomography (CT) scan of the knee showing an AO 41 B3.1 fracture type.

Patients between the age of 18 and 65 years with a solitary, acute, traumatic, closed depression fracture of the proximal tibia (limited to AO types 41 B2 & B3, Schatzker types II and III) (Figures 2, 3, and 4) requiring reconstruction of the subchondral bone void are enrolled into the trial. The ability to understand the procedure of participating in the study and to comply with the follow-up program is a prerequisite. Only patients with a maximum interval of one week between injury and intervention will be included.

Patients will be excluded from the study if the responsible surgeons request treatment in which the principle of therapeutic uncertainty is violated. Clinical exclusion criteria involve patients with more than one injury, polytrauma patients, compartment syndrome, previous iliac crest bone graft harvesting, local infection at the site of implantation, chronic pain disease, malignancy, rheumatoid arthritis, chronic cortisone therapy, unavailable X-ray diagnostics, doubtful fracture classification, and clinically significant unstable medical or surgical conditions that may preclude safe and complete study participation.

Device-related exclusion factors are pre-existing calcium metabolism disorders (for example, hypercalcemia), hyperthyroidism, or autonomous thyroid adenoma, a history of serious reaction to iodine-based radio contrast agents, pregnancy or breastfeeding (a pregnancy test will be performed at screening visit), irreversible coagulopathy or bleeding disorders, history of physical or psychological condition that contraindicates the use of an investigational device or render the patient at high risk from treatment complications, as well as history of hypersensitivity to the device or any of its ingredients. Participation in other clinical trials during the present clinical trial or within the last month will be also considered an exclusion criterion.

### Randomization

After giving consent to participate in the study, patients will be randomized by a web-based randomization tool provided by IZKS Mainz allowing investigators to randomize patients via a secure web interface. Randomization will be stratified by age group (18 to 39 years; 40 to 65 years) and gender. Block randomization with variable block sizes will be applied.

### Interventions

All fractures will be immobilized with a plaster cast until surgical treatment. Centers may follow their preferred standard operating procedures and locally established protocols for pain management. These modalities will be precisely documented.

### Operative treatment

**Figure 5:**
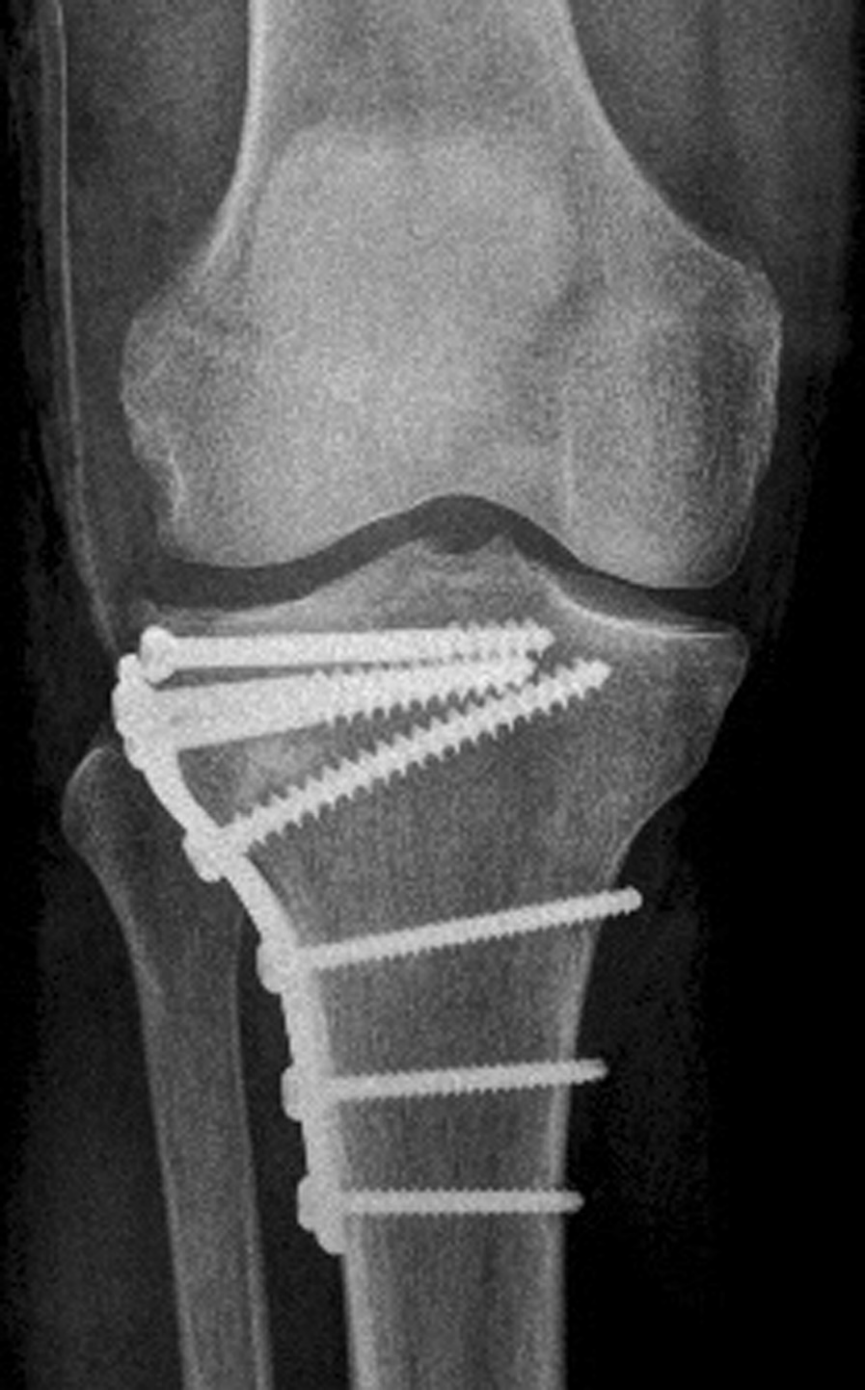
**Postoperative anteroposterior (a-p) X-ray of the knee showing the result of reconstruction.** The subchondral bone defect was filled with CBVF.

**Figure 6:**
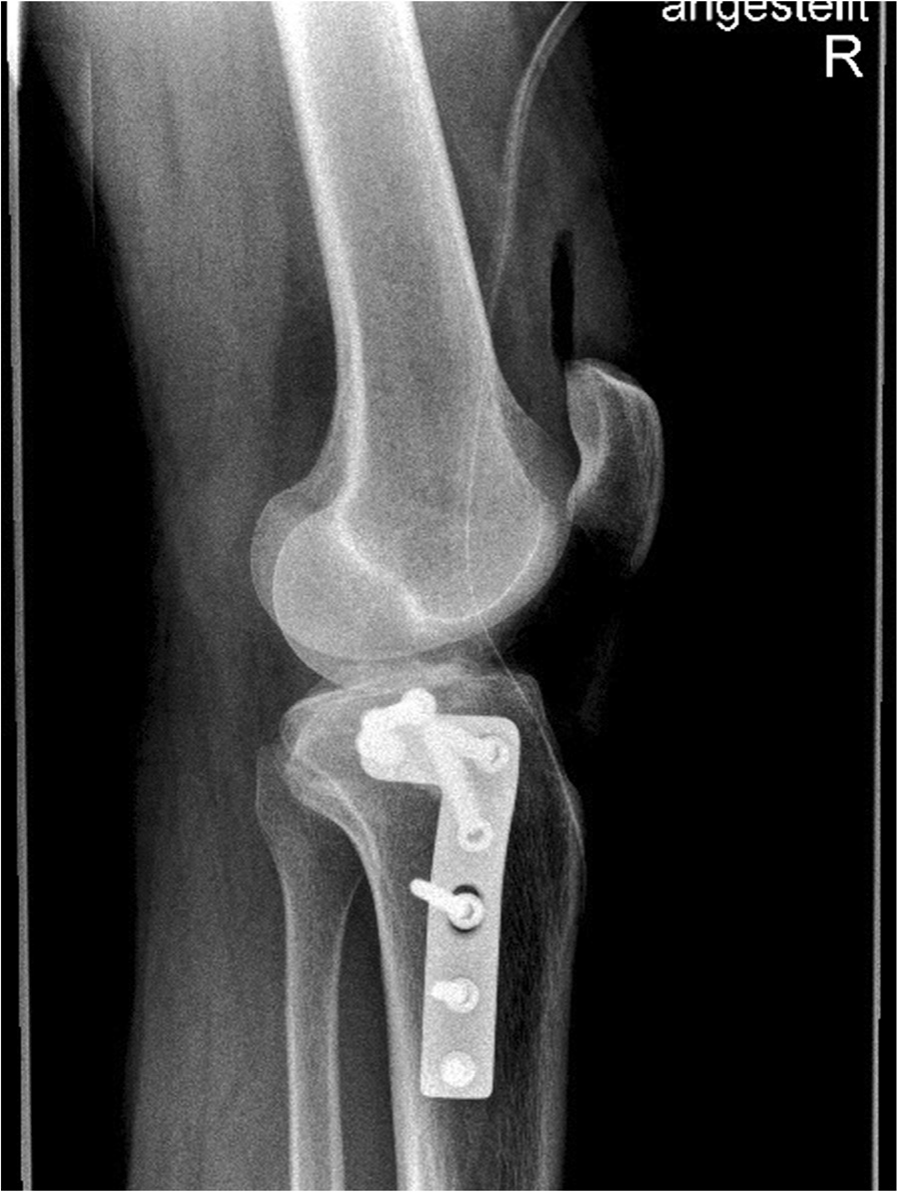
Postoperative lateral X-ray of the knee showing the reconstruction result.

After randomization, all patients will be operated as soon as possible, depending on local soft tissue conditions, hematoma, and swelling. Open reduction and internal fixation will be performed via an anterolateral approach using screws and buttress plate for stabilization after reduction of the depressed articular surface according to the AO principles of fracture care. The protocol does not specify the use of specific hardware. The bone defect, which is created after reduction of the depressed articular fragments, will be reconstructed either with an autologous cancellous bone graft from the iliac crest or with the CBVF (Figures 5 and 6). All surgeons performing the operation have proven expertise with the procedure and are familiar with the implants and devices used in this study.

### Outcome measures

#### Primary outcome

The primary endpoint in this study is the SF-12 v2 Physical Component Summary (PCS) score (4 week standard recall version) at week 26 after the intervention. The co-primary endpoint is pain VAS measurement at week 26 after the intervention. The SF-12 is a subset from items of the SF-36, which is the most widely used generic health assessment questionnaire worldwide.

#### Secondary outcomes

The SF-12v2 Mental Component Summary (MCS) at week 26 and costs of care related resources will be evaluated as key secondary endpoints.

Services relevant to cost of care will be estimated from the type of insurance (private or compulsory), days spent in the hospital as the result of the trauma, total costs of care for the hospital stay in Euro, DRG (diagnosis-related group) code and the number of lump compensations for ambulant treatment. In addition, the patient will be asked to document the costs of care for services related to trauma and applied outside the trial center (for example, physiotherapy, medical aids) beginning with the day of discharge from the trial center. The assessment of costs will be specific for local German conditions as this study will be performed in German centers only.

#### Other endpoints

SF-12v2 PCS and SF-12v2 MCS will be assessed as additional endpoints at visits 4 to 6, the pain measured by VAS (visits 3 to 6) as well as the intake of pain medication at visits 3 to 7. Fracture healing and integrity of the articular surface will be assessed using standard anteroposterior and lateral views of plain radiographs. The radiographs will be reviewed by the panel of two experienced orthopedic trauma surgeons and one consultant who will be blinded as to the kind of material used. The panel will independently review all radiographs in chronological order. The loss of reduction and subsidence of the articular surface will be recorded and analyzed using the Rasmussen score [17], in which articular subsidence, condylar widening, and varus or valgus deviation will be quantitatively assessed over a period of 26 weeks. In addition, the panel will evaluate fracture union, bone defect remodeling as well as loss or premature resorption of the graft using the following point score:

1. Osteolysis, premature resorption of the graft

2. Unchanged size of the void, no resorption of the bone graft

3. Beginning marginal bone defect remodeling

4. Bone defect remodeling with undirected formation of trabecula

5. Bone defect remodeling with directed formation of trabecula

Complications (for example, hardware failure and displacement, wound healing problems, infections, revisions, neurologic impairment, pressure ulcers and others) as well as all adverse events (AE) will be recorded and regularly evaluated throughout the study.

#### Evaluation and follow-up

Clinical evaluation and trial documentation consist of seven visits: screening (V1), intervention (V2) and five postoperative follow-up examinations ending with V7 after 26 weeks (Table 1).

**Table 1:** Visit schedule

**Visit no**	**1**	**2**	**3**	**4**	**5**	**6**	**7**^**§**^
	**Day −7 – 0**	**Day 0**	**Day 1**	**Day 7**	**Week 6**	**Week 12**	**Week 26**
**Visit window**	**Screening**	**Day of surgery**	**± 1 day**	**± 3 days**	**± 1 weeks**	**± 2 weeks**	**± 3 weeks**
Informed consent	X						
Inclusion/Exclusion criteria	X						
Demography	X						
Patient employment status	X						
Medical history	X						
Weight and Height	X						
Physical examination	X						
Vital signs (pulse, blood pressure)	X						
Pregnancy test (women of childbearing potential)	X						
Concomitant medication/therapy	X	X	X	X	X	X	X
Evaluation of CT scan*	X						
Evaluation of X-ray findings*	X		X	X	X	X	X
Randomization	X						
Surgery** (autologous bone graft or treatment with CBVF***)		X					
Procedure information (type and amount of bone graft, hardware used, etc. - time of surgery)		X					
Adverse events	X	X	X	X	X	X	X
Device complaints		X	X	X	X	X	X
Clinical examination (pain, tenderness, warmth and swelling)				X	X	X	X
Documentation of treatments performed outside the trial center****							
Pain medication	X	X	X	X	X	X	X
SF-12v2TM	X			X	X	X	X
Pain (VAS)	X		X	X	X	X	X
End of the trial							X

*Computed tomography (CT) and X-ray assessments are performed as a clinical routine according to the standards of care. They will be not performed for the purposes of the study. **Surgical procedures are performed according to the standard of care. No surgical interventions will be performed for the purposes of the study. ***CBVF, CERAMENT™|BONE VOID FILLER. ****Continuous documentation by patient, when applicable. §These examinations should also be performed at the trial end visit in case the patient prematurely drops out of the trial.

#### V1 - screening visit and randomization

The trial aims and procedures will be explained to the patient by responsible local investigators using a study information booklet. After informed consent is given, the screening visit (V1) will be performed within 7 days prior to day of surgery (day 0). After radiological assessment of the fracture type, baseline demographics, comorbidity, medication, and pregnancy test (if applicable) will be evaluated and verification of inclusion and exclusion criteria performed. The trial participant will be electronically randomized to one of the two treatment arms using a web-based tool. All data will be documented using an electronic case report form (eCRF). Assessment of the VAS and SF-12v2 scores will be performed at the end of the V1.

#### V2 - intervention

This visit is intended to document the surgical procedures and procedure-related complications. In both treatment arms the responsible surgeon performs the intervention. The test device is administered according to the instruction for use (IFU) for CBVF. Fracture surgery and, if applicable, harvesting of autologous bone graft will be performed according to the well-established standards of care.

After the surgery, information on the type of surgery, osteosynthesis material, volume of CBVF or autologous cancellous bone graft are documented in the eCRF. Furthermore, adverse events including the device complaints will be documented as well. Postoperative care is performed according to the local SOPs.

#### V3 - day 1 post intervention

Radiographs are taken between day 1 to day 3 to evaluate the quality of reduction of the articular surface as well as the appropriate positioning of the hardware and the device. Furthermore, adverse events including device complaints, the VAS score, and concomitant medication including pain medication will be documented in the eCRF.

#### V4 - day 7 post intervention

The following procedures will be performed: evaluations of the X-rays, monitoring of adverse events including device complaints, the SF-12v2 score, VAS, concomitant medication including pain medication, and clinical healing evaluation (pain, tenderness, warmth, and swelling).

#### V5 - 6 weeks post intervention and V6 - 12 weeks post intervention

Patients will be evaluated in an outpatient clinic by the responsible surgeon. Results of the radiological assessment which is required according to the standard of care will be documented in the eCRF. Possible local complications such as pain, tenderness, warmth, and swelling will be assessed. The number of physiotherapy courses and the need for other therapeutic interventions will be documented in the patient’s diary. Adverse events including device complaint monitoring, the SF-12v2 score, VAS, and concomitant medication including pain medication will be recorded.

#### V7 - 26 weeks post intervention, final visit

The final visit will be performed six months after the intervention. The results of the clinical and radiological evaluation as well as the adverse events and device complaints will be documented in the eCRF. The SF-12v2 score, the VAS score and medication will be assessed and recorded. Computed tomography scans (CTs) and x-rays will be sent anonymously to coordinating investigator for central blinded evaluation.

### Statistics

#### Pre-specification

Details of the statistical analysis of the data collected in this trial will be documented in a Statistical Analysis Plan (SAP) that will be generated by IZKS Mainz and finalized before closing the database. The SAP is based on the protocol including all amendments. The document may modify the plans outlined in this protocol; however any major modifications of the primary endpoint definition and/or its analysis will also be reflected in a protocol amendment. Any deviation from the original SAP must be described and justified in the final report. The statistical analysis will be conducted by means of SAS™.

#### Sample size and power calculation

Sample size was calculated for the primary endpoint (SF-12v2 PCS week 26). Additional power calculations were performed for the co-primary endpoint (VAS pain score week 26). Within the German norm population, mean SF-12v2 PCS is 50 and the standard deviation is 10 [18]. The study population is expected to come very close to this German norm population. Based on the inclusion criteria (age between 18 and 65) and demographic data of the proximal tibia fractures treated at the trial site in Mainz between 2009 and 2011, it seems justified to expect the SF-12v2 PCS results 26 weeks after surgery to be 50 ± 10 (mean ± standard deviation) in both treatment groups. The non-inferiority margin was defined as 5 points on the SF-12v2 PCS scale (range: 0–100). This value is half of the expected standard deviation in the reference population. Since changing one of the SF-12v2 items affecting SF-12v2 PCS by one category causes SF-12v2 PCS to change by at least 3 points, the non-inferiority margin corresponds to less than two items changed by one response category. Under these assumptions, a shifted two-sample t-test with a one-sided significance level of 2.5% and a power of 80% requires 128 patients to show that mean SF-12v2 PCS 26 weeks after surgery in patients treated with CBVF is not more than 5 points lower compared to the control group. Since 5% of randomized patients are expected to be ineligible for the primary Per Protocol Analysis, 136 patients will be randomized. Pain assessed by VAS after 26 weeks is expected to be 2 (80%) or 1 (20%) in the control group and 2 (10%), 1 (20%) or 0 (70%) in the CBVF treatment group. Based on these assumptions, the probability that pain assessed on a VAS comparing one randomly selected patient in each group is higher in the patient treated with CBVF is 14%. A Wilcoxon-Mann–Whitney-Test comprising 128 patients will have a power of >99% to show at a two-sided significance level of 5% that the above specified probability is <50%.

### Analysis populations

The intention to treat (ITT) population comprises all randomized patients, even if the planned surgery/surgeries did not take place. The modified intention to treat (mITT) population includes all randomized patients for which the primary endpoint could be assessed (that is, SF-12 PCS assessment is available both preoperatively and 26 weeks after surgery). Patients are eligible for the per protocol (PP) population, if they fulfill the following criteria: eligible for the mITT population, no violation of inclusion criteria, not meeting any exclusion criteria, trial treatment as randomized, time interval between Visit 2 (day 0, surgery) and Visit 7 (week 26): 22 to 30 weeks, absence of events with a strong effect on quality of life but definitely unrelated to the randomized study treatment (for example, new serious disease during post-operative follow-up, or accident).

The safety population comprises all randomized subjects who received trial treatment (autologous bone graft or Treatment with CBVF). In analyses of the safety population, subjects will be assigned to the treatment they actually received.

### Efficacy analysis

Within the primary analysis of this study, SF12v2 summary scores are not normalized to the reference population. Nevertheless, normalized z and t scores of SF12v2 summary scores will be calculated in explorative analyses. SF-12v2 standard 4-weeks recall version will be used within this study. The confirmatory efficacy analysis is planned using a hierarchical testing procedure. In the first step, differences between SF-12v2 PCS are tested by an analysis of covariance (ANCOVA) with SF-12v2 PCS at week 26 as the dependent variable and SF-12v2 PCS at screening, age group (18–39; 40–65), gender, and treatment as covariates. If the covariate-adjusted two-sided 95% confidence interval of the treatment effect within this model (difference of SF-12v2 PCS, experimental treatment group - control group) is located completely above the non-inferiority margin of −5, non-inferiority of CBVF with respect to Physical Component of Quality of Life 26 weeks after surgery will be claimed. The primary endpoint will be primarily analyzed in the PP population. Only if non-inferiority can be claimed in the first step, pain as the co-primary endpoint may be tested in a confirmatory way. Differences between pain assessed by VAS 26 weeks after surgery will be tested by a Wilcoxon-Mann–Whitney-U-Test at a two-sided significance level of 5%. Analysis will be done primarily for the mITT population. Within this hierarchical testing procedure, a global significance level of 5% is ensured. The power was not adjusted since the local power of the second test (pain assessment) is sufficiently large (>99%). In order to check robustness of confirmatory results, supportive analyses of both (co-)primary endpoints will be performed in the mITT, PP and ITT populations. Models with a modified set of covariates (for example, trial site and surgical technique) will also be calculated as supportive analyses. Analyses in the ITT population will require imputing missing data. Multiple imputation methods are planned within this context. Further details will be specified in the Statistical Analysis Plan. All secondary endpoints will be analyzed using appropriate methods depending on the scale of the respective parameter. Analyses of secondary endpoints will be interpreted exploratively.

### Safety analysis

All summaries and listings of safety data will be performed for the safety population. Frequencies of subjects experiencing at least one adverse event (AE) will be displayed by body system and preferred term according to MedDRA terminology. Detailed information collected for each AE will include: a description of the event, duration, whether the AE was serious, intensity, relationship to trial treatment, action taken, and clinical outcome. Summary tables will present the number of subjects observed with AEs and corresponding percentages. Additional subcategories will be based on event intensity and relationship to trial treatment. Analysis of device (CBVF)/graft (autologous bone) complaints will be analogously as far as possible.

### Withdrawal criteria

Patients can withdraw their consent without giving reasons for withdrawal at any time point of the study. The withdrawal will not have any negative consequences for the participant. The participation in this study may be discontinued:

1. upon the request of the sponsor for safety reasons

2. upon the request of a regulatory agency

3. if serious adverse events related to the therapy occurred

4. in case of non-compliance of participant

5. if participation in the trial would be of potential hazard to study participant´s health

6. if study participant will need a treatment which is specified in the protocol as exclusion criterium

7. if study participant becomes pregnant

Participants withdrawn from the study will not be replaced regardless of the reason for withdrawal. The investigator decides about the withdrawal of study until resolve of symptoms, but no longer than 6 months after the subject’s withdrawal from the trial.

### Assessments of safety

All adverse events (AE) and serious adverse events (SAE), including device-related events will be assessed und documented. Some AE may lead to secondary surgical interventions. According to the Good Clinical Practice (GCP) guidelines, secondary surgical interventions will be categorized as follows:

1. A revision is a procedure that adjusts or in any way modifies or removes part of the original implant configuration, with or without replacement of component. A revision may also include adjusting the position of the original configuration.

2. A removal is a procedure where the entire original system configuration is removed with or without replacement.

3. A reoperation is any surgical procedure at the involved site that does not remove, modify or add any components to the system.

4. A supplemental fixation is a procedure in which additional instrumentation not under study in the protocol is implanted (for example, supplemental placement of a rod/screw system or a plate/screw system).

5. All these secondary surgical interventions will be considered serious adverse events.

According to the Food and Drug Administration (FDA) definition, a device complaint is any written, electronic or oral communication that alleges deficiencies related to the identity, quality, durability, reliability, safety effectiveness, or performance of a device. All kinds of AEs (whether serious or non-serious) reported by the subject or detected by the investigator will be documented in the eCRF and in the patient´s medical records.

### Trial documentation and data collection

A detailed methodology for the data management in this trial will be documented in a data management plan (DMP) that will be dated and maintained by IZKS Mainz. The data acquisition will be performed using a web-based electronic case report form (eCRF). The investigator will enter the data via remote data entry (RDE) directly into the trial database, developed and maintained by IZKS Mainz. The system will be secured to prevent unauthorized access to the data or the system. The system provides the option of making exact data copies in legible paper form for review and audits. The investigator or a designated sub-investigator, following review of the data in the eCRF, will confirm the validity of each subject’s data by electronic signature or by signing a paper printout of all trial´s data. Checks for plausibility, consistency, and completeness of the data will be performed during and after data entry. After completion of data entry and if no further corrections are to be made in the database, the access rights will be taken away and the database will be declared closed and used for statistical analysis. All data management activities will be done according to the current standard operating procedures (SOPs) of IZKS Mainz. The names of the study participants and all other confidential information will be handled according to the medical professional secrecy and the regulations of the German Federal Data Protection Act. None of the patient-related information will become available to any person who is not directly involved in the medical treatment. During the clinical trial, subjects will be identified solely by means of an individual identification code. All trial results stored on a computer will be handled in accordance with the local data protection law in strictest confidence. For protection of these data, organizational procedures are implemented to prevent distribution of data to unauthorized persons. The appropriate regulations of data legislation will be fulfilled in its entirety. The subject will declare in the written consent to release the investigator from the medical professional secrecy to allow identification of subject’s name and/or inspection of original data for monitoring purposes by authorities and authorized persons.

### Source of bias

To reduce ascertainment bias, QOL and pain assessments will be performed by independent surgeons who will not be directly involved in the operative treatment of the patients. Furthermore, the assessments will be performed by persons with broad experience in treating trauma patients to determine whether or not events impacting QOL are really related to the treatment. The assessors will document all relevant events potentially impacting QOL and pain in the trial report. However, a consequent blinding of the assessors will not be feasible, since an additional wound will be obvious in all patients of the autologous bone graft group. Therefore, the method of assessment may be associated with an ascertainment bias, which cannot be avoided completely by the study design.

### Ethical issues

The procedures set out in the trial protocol, pertaining to the conduct, evaluation, and documentation of this trial, are designed to ensure that all persons involved in the trial abide by Good Clinical Practice [19] and the ethical principles described in the Declaration of Helsinki [20]. The trial is carried out in compliance with local legal and regulatory requirements. Prior to enrollment of the first subject, all ethical and legal requirements will be met by inclusive unreserved approval by institutional review boards. The final study protocol and the final version of the written informed consent form were already approved by the ethics committee of the University of Mainz, which is responsible for the site of the principal investigator. Medical experts have performed the critical assessments of risks and benefits in advance. Each site’s principal investigator ensures that all persons assisting with the trial are adequately informed about the protocol, the trial treatments, and their trial related duties and functions. Before being enrolled into the clinical trial, each subject must consent to participate after nature, scope, and possible consequences of the clinical trial have been explained to him or her in an understandable oral and written form. The subject must give consent in writing or orally in presence of an independent witness before randomization. Results of study will be published according to the most recent version of the Consolidated Standards of Reporting Trials statement [21].

## Discussion

Autogenous cancellous bone from the iliac crest is the most frequently used site for bone-graft harvest and remains to be the 'gold standard’ in reconstruction of bone defects and fracture non-unions in both metaphyseal and diaphyseal areas. The co-morbidities related to harvesting of autologous bone grafts are well documented [4,22-26]. Bone substitutes are used to avoid donor site problems associated with the harvest of the iliac crest bone graft. The clinical and functional outcome when comparing the use of autologous bone graft with the use of a bone graft substitute has been investigated in several studies [8,13,22,27]. However, the endpoints of those studies were mostly related to the ‘quality’ of the surgery performed. Good clinical outcome is directly related to the quality of the anatomical reduction of tibial plateau fractures [13,28]. Tibia plateau fractures have a high incidence of reduction loss when fixed without augmentation of the depressed articular fragments [4,29]. Actually, there are no studies that compare pain, QoL, and costs of care, which are all relevant endpoints related to the bone graft used. This necessitates the investigation of these outcomes in a sufficiently powered, prospective, randomized, controlled clinical trial. Current evidence, specifically reliable information from RCTs, is insufficient to counsel patients and their relatives with regard to the most convenient, efficient, and safe method to avoid prolonged pain and to restore a high level of quality of life and activity. As far as we know, the CERTiFy trial is the first randomized multicenter RCT that aims at generating conclusive evidence on the most appropriate way of treating tibia plateau fractures.

## Trial status

Recruiting

## Abbreviations

AE: adverse event; AIBG: autologous iliac bone graft; ANCOVA: analysis of covariance; AO: Arbeitsgemeinschaft Osteosynthese; CBVF: CERAMENT bone void filler; CE: Communautés Européennes; CERTiFy: CERAMENT treatment of fracture defects; CPC: calcium phosphate cements; CRF: case report form; CS: calcium sulfate; CT: computed tomography; DMP: data management plan; DRG: diagnosis related group; eCRF: electronic case report form; FDA: Food and Drug Administration; GCP: good clinical practice; HA: hydroxyl apatite; IFU: instruction for use; ITT: intention to treat; IZKS: Interdisziplinäres Zentrum für Klinische Studien Mainz; MCS: Mental Component Score; mITT: modified intention to treat; ORIF: open reduction and internal fixation; PCS: Physical Component Summary; PP: per protocol; Qol: quality of life; RCT: randomized controlled trial; RDE: remote data entry; SAE: serious adverse event; SAP: statistical analysis plan; SAS: statistical analysis system; SF-12v2: Short Form 12 Version 2; SF-12v2-PCS: Short Form 12 Version - physical component score; SF12v2-MCS: Short Form 12 Version 2 - mental component score; SF-36: Short Form 36 score; SOP: standard operating procedure; VAS: Visual Analog Scale.

## Competing interests

The authors declare that they have no competing interests.

## Authors’ contributions

TN wrote the manuscript, reviewed the study design, performs data acquisition and management, revised the manuscript. AH coordinating trial investigator, wrote the manuscript, initiated and designed the study, performs data acquisition and management, coordination between study centers, revised the manuscript. DW responsible trial biostatistician, revised the manuscript. SG participated in developing the trial design and trial protocol and coordinates multi-center management, revised the manuscript. PMR responsible trial investigator, initiated and designed the study, revised the manuscript, coordination between study centers. All authors read and approved the final manuscript.
